# Africans Who Arrive in the United States before 20 Years of Age Maintain Both Cardiometabolic Health and Cultural Identity: Insight from the Africans in America Study

**DOI:** 10.3390/ijerph17249405

**Published:** 2020-12-15

**Authors:** Elyssa M. Shoup, Thomas Hormenu, Nana H. Osei-Tutu, M. C. Sage Ishimwe, Arielle C. Patterson, Christopher W. DuBose, Annemarie Wentzel, Margrethe F. Horlyck-Romanovsky, Anne E. Sumner

**Affiliations:** 1 Section on Ethnicity and Health, Diabetes, Endocrinology, and Obesity Branch, National Institute of Diabetes and Digestive and Kidney Diseases, Bethesda, MD 20892, USA; elyssa.shoup@nih.gov (E.M.S.); thormenu@ucc.edu.gh (T.H.); nana.osei-tutu@nih.gov (N.H.O.-T.); sishimwe@ughe.org (M.C.S.I.); pattersonarc@niddk.nih.gov (A.C.P.); christopher.dubose@nih.gov (C.W.D.); annemarie.wentzel.aw@gmail.com (A.W.); 2 National Institute of Minority Health and Health Disparities, Bethesda, MD 20892, USA; 3 Institute of Global Health Equity Research, University of Global Health Equity, Kigali 6955, Rwanda; 4 Department of Health and Nutrition Sciences, Brooklyn College, City University of New York, New York, NY 11210, USA; MargretheHR@brooklyn.cuny.edu

**Keywords:** African immigrants, cardiometabolic health, socioeconomic status, stress

## Abstract

The overall consensus is that foreign-born adults who come to America age < 20 y achieve economic success but develop adverse behaviors (smoking and drinking) that lead to worse cardiometabolic health than immigrants who arrive age ≥ 20 y. Whether age of immigration affects the health of African-born Blacks living in America is unknown. Our goals were to examine cultural identity, behavior, and socioeconomic factors and determine if differences exist in the cardiometabolic health of Africans who immigrated to America before and after age 20 y. Of the 482 enrollees (age: 38 ± 1 (mean ± SE), range: 20–65 y) in the Africans in America cohort, 23% (111/482) arrived age < 20 y, and 77% (371/482) arrived age ≥ 20 y. Independent of francophone status or African region of origin, Africans who immigrated age < 20 y had similar or better cardiometabolic health than Africans who immigrated age ≥ 20 y. The majority of Africans who immigrated age < 20 y identified as African, had African-born spouses, exercised, did not adopt adverse health behaviors, and actualized early life migration advantages, such as an American university education. Due to maintenance of cultural identity and actualization of opportunities in America,**** cardiometabolic health may be protected in Africans who immigrate before age 20. In short, immigrant health research must be cognizant of the diversity within the foreign-born community and age of immigration.

## 1. Introduction

Between 2010 and 2018, immigration from sub-Saharan Africa to the United States increased by 52% [1]. This is four times higher than the 12% increase in the overall rate of immigration to the United States [1]. African immigrants are well-educated and contribute to the workforce at a higher rate than other immigrant groups or native-born Americans [1,2], yet little is known about the cardiometabolic health of African immigrants. In addition, there is a paucity of data on how factors, such as age of immigration and cultural transition, influence their cardiometabolic health. 

In adapting to life in the United States, African immigrants have to face many challenges, including the transition from a majority to a minority population and racial discrimination [1,3,4]. In short, international migration has both physiologic and psychological consequences and the potential to adversely affect cardiometabolic health [3,4,5,6,7,8]. Cardiometabolic health encompasses obesity, diabetes, dyslipidemia, and cardiovascular disease (CVD). CVD is a composite term for coronary heart, cerebrovascular and peripheral artery diseases, and heart failure [9].

While data on the health of African immigrants are scant and largely limited to self-report [4,10], extensive data on Hispanic immigrants are available [5,6,11,12,13]. Hispanic immigrants who arrive in the United States before age twenty are more likely to achieve English-proficiency, higher education, and economic prosperity than older immigrants [12]. However, immigrants who come to the United States at a young age are also more likely to smoke, drink alcohol and develop obesity than older immigrants [5,6,11,12,13]. Therefore, in adulthood the mortality rate for Hispanic immigrants who arrived in the United States before age 20 is higher than for those who arrived after 20 years of age [5,12].

For African immigrants, the influence of generation, behavior, and social conditions on cardiometabolic health as assessed by obesity status, diabetes prevalence, and cardiovascular risk are unknown. In addition, African region of origin and francophone status of birth country are rarely considered when evaluating the health of African immigrants to the United States. Therefore, we worked with the Africans in America cohort and collected cultural, socioeconomic, and immigration-specific data. The cultural and behavioral factors studied were ethnic identification, marriage to an African-born spouse, physical activity, smoking, and alcohol intake. Socioeconomic factors investigated were education, location of university attended, income, and health insurance. Immigration-specific factors included age at immigration, duration of residence in the United States, and African region and francophone status of the country of origin.

Our goals were to examine cultural identity, behavior, and socioeconomic factors and determine if there are differences in the cardiometabolic health of Africans who immigrated to the United States at less than 20 years of age (Immigration-Age < 20 y) compared to those who came to the United States at 20 years of age or greater (Immigration-Age ≥ 20 y).

## 2. Materials and Methods

The Africans in America cohort was designed to assess the cardiometabolic health of African-born Blacks living in the United States [14,15,16,17]. At enrollment, all participants were between the ages of 20 and 65 years. Recruitment was achieved with newspaper advertisements, announcements on the NIH website, flyers, presentations at community events, and referrals by previous participants. The protocol was approved by the NIDDK Institutional Review Board (Clinical Trials.gov Identifier: NCT00001853). Every participant gave written informed consent.

Telephone interviews were conducted prior to enrollment. Potential participants had to state that they self-identified as Black, were born in a sub-Saharan African country, and that their parents also self-identified as Black and were born in a sub-Saharan African country. In addition, potential participants had to self-identify as healthy and deny a history of diabetes.

Five hundred and twenty-two African-born Blacks living in metropolitan Washington, DC, completed the telephone interview and proceeded to Visit 1, which was an outpatient screening visit conducted at the NIH Clinical Center, Bethesda, Maryland. At the screening visit, social and medical histories were taken, and a physical examination, an electrocardiogram, and routine blood tests were performed. Forty individuals did not proceed from Visit 1 to Visit 2. Reasons for not proceeding to Visit 2 included screening laboratory identification of anemia, elevated liver transaminases, hypothyroidism, intravenous access issues, or scheduling challenges.

As a result, 482 enrollees proceeded to Visit 2, which occurred 10 ± 8 days after Visit 1. At Visit 2, weight, height, blood pressure (BP), waist circumference (WC), a fasting lipid profile, A1C, and hemoglobin electrophoresis were measured, and an oral glucose tolerance test (OGTT) (Trutol 75, Custom Laboratories) was performed.

### 2.1. BMI Status 

BMI was defined according to standard criteria (Normal weight: BMI 18.5 to 24.9 kg/m^2^; Overweight: BMI 25.0 to 29.9 kg/m^2^; Obesity: BMI ≥ 30 kg/m^2^) [18]. 

### 2.2. Diabetes

Diabetes was diagnosed if A1C ≥ 6.5% or glucose criteria for the OGTT were met (fasting glucose ≥ 126 mg/dL and/or 2 h glucose ≥ 200 mg/dL) [19].

### 2.3. Determination Cardiovascular Disease Risk Score 

We used the 10-year Framingham Cardiovascular Disease Risk Score (CVD-Risk) because it was validated in African Americans and calculated based on a formula that used sex-specific thresholds for six parameters: age, smoking, systolic BP, total cholesterol, high density lipoprotein (HDL) cholesterol, and diabetes [9,20].

### 2.4. Social Variables

Data for all participants were available for five variables which were dichotomized: education (college graduation rate), income (≥40 k/year), smoking (Yes or No in the last year), alcohol intake (one or more drinks per week vs. less than once per week), and marital status. Marital status was defined as partnered (married or living with partner) or not partnered (never married, separated, divorced, or widowed) [4]. For duration of residence in the United States, data were missing for four individuals (*n* = 478). For five variables, data were collected consecutively from the most recently enrolled participants. The number of participants with data for each of these variables were physical activity (*n* = 270), self-categorization (African or African-American) (*n* = 255), ethnicity of spouse (*n* = 220), and location of university attended (United States, Africa or elsewhere) (*n* = 74). For the 220 individuals who were enrolled after the question about ethnicity of partner was initiated, 51% (113/220) were partnered. Exercise intensity was determined by International Physical Activity Questionnaire categories and dichotomized as sedentary (IPAQ category Low) or active (IPAQ category Moderate or High) [21].

### 2.5. Assays

Hemoglobin and hematocrit were measured in EDTA-anticoagulated whole blood using a Sysmex XE-5000. Glucose, cholesterol, triglyceride, HDL, LDL, apolipoprotein A1 (apoA1), and apolipoprotein B (apoB) were measured in lithium–heparin plasma (Roche/Hitachi Cobas C, Roche Diagnostics, Indianapolis, IN, USA). A1C levels were determined by HPLC using National Glycohemoglobin Standardization Program (NGSP) [22]. Identities of hemoglobin proteins were confirmed by comparison with known samples of HbA, HbS, and HbC.

### 2.6. Statistical Analyses

Unless stated otherwise, data are presented as mean ± SE. The cohort was divided by immigration age into two groups: arrival before the age of 20 (Immigration-Age < 20 y) and arrival at age 20 or older (Immigration-Age ≥ 20 y). For sub-analyses, the Immigration-Age < 20 y group was further divided into two groups: Immigration-Age < 10 y and Immigration-Age-10-to-19-y. Comparisons were by unpaired *t*-tests or one-way ANOVA with Bonferroni correction for continuous variables and by chi-square tests for categorical variables. Comparisons of the two immigration groups of all biological parameters, including BMI, WC, blood pressure, lipids, and CVD-Risk score, were adjusted for age.

In addition, multiple linear regressions were performed separately for the Immigration-Age < 20 y and Immigration-Age ≥ 20 y groups. The dependent variable was CVD-Risk, and the independent variables were age of immigration, duration of US residence, health insurance, education, income, and alcohol intake. Smoking was not included as an independent variable as smoking status is included in the calculation of the CVD-Risk [9]. For the Immigration-Age ≥ 20 y group, duration of United States residence was divided into five-year increments (0 to 4 y, 5 to 9 y, 10 to 14 y, 15 to 19 y and 20 years plus). For the Immigration-Age < 20 y group, duration of United States residence intervals were: 0 to 9 y, 10 to 14 y, 15 to 19 y, and 20 years plus. The 0 to 4 y and 5 to 9 y intervals were combined, because there were only two individuals in the Immigration-Age < 20 y group who had been in the United States for less than five years.

*p*-Values ≤ 0.05 were considered significant. Data were managed with Research Electronic Data Capture (REDCap) [23]. Analyses were performed with STATA16 (College Station, TX, USA).

## 3. Results

The 482 participants (male: 65%; age: 38 ± 0.1 y, range 20 to 65 y; BMI: 27.6 ± 0.2 kg/m^2^, range 18.2 to 42.4 kg/m^2^) were from four areas of sub-Saharan Africa: West (52%, 253/482), Central (20%, 96/482), East (28%, 133/482), and South (<1%, 4/482). The four participants from Southern African countries were included in the Central African group (Supplement Table S1). Comparison by African region of origin revealed no differences in socioeconomic, cardiometabolic, or cultural factors including gender, age, age at immigration, or frequency of self-identification as African. However, West Africans had the longest residence in the United States. In addition, heterozygous hemoglobinopathies (i.e., sickle cell trait and hemoglobin C trait) were more common in West and Central Africans than East Africans.

**Figure 1:**
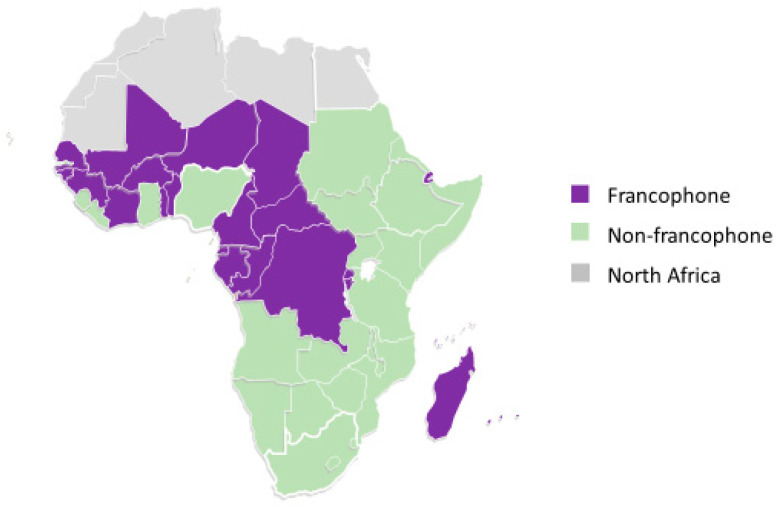
Geographical Distribution of Sub-Saharan African Countries by Francophone Status.

When the cohort was analyzed according to whether the country of origin was francophone (34%, 162/482) or non-francophone (66%, 320/482) (Figure 1), immigrants from francophone countries were older at immigration (29 ± 1 vs. 25 ± 1 y, *p* = 0.002) and had been in the United States for a shorter duration (10 ± 1 vs. 13 ± 1 y, *p* < 0.001) (Supplement Table S2).

However, as with geographic region, there were no significant differences in cultural identity, behavior, and socioeconomic factors. In addition, differences by immigration age group were similar in the francophone and non-francophone subgroups (Supplement Tables S3 and S4). Therefore, the cohort was analyzed without regard to African region of origin or francophone status of birth country.

### 3.1. Age, Temporal and Gender Characteristics

Twenty-three percent (111/482) of participants were in the Immigration-Age < 20 y group and 77% (371/482) in the Immigration-Age ≥ 20 y group (Table 1). 

**Figure 2:**
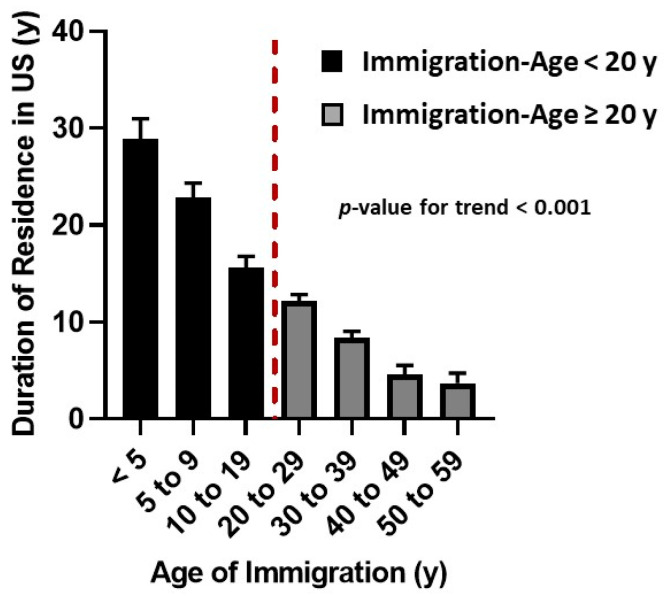
Duration of Residence in the United States by Age of Immigration. Dash red line separates the group who immigrated before age 20 y from the group who immigrated at 20 y or older.

Participants who came before the age of 20 y were younger (31 ± 1 vs. 40 ± 1 y, *p* < 0.001) and had been in the United States for longer (24 ± 1 vs. 8 ± 1 y, *p* < 0.001) (Table 1) (Figure 2). 

Gender distribution differed by immigration age group, as percent male in the Immigration-Age < 20 y group was lower than the percent male in the Immigration-Age ≥ 20 y group, (53% vs. 69%, respectively) (*p* = 0.003). 

### 3.2. Cardiometabolic Health

In the overall cohort, there was no significant difference by immigration age group in prevalence of obesity, diabetes or CVD-Risk. However, for men, the lipid profile was better for the Immigration-Age < 20 y group than the Immigration-Age ≥ 20 y group. Specifically, both HDL and apoA1 were higher in the Immigration-Age < 20 y group than the Immigration-Age ≥ 20 y group (*p* = 0.014 and *p* = 0.015, respectively), (Table 1). In addition, triglyceride concentration tended to be lower in the Immigration-Age < 20 y group (*p* = 0.062) (Table 1). 

### 3.3. Social Factors

#### Cultural Identity

**Figure 3:**
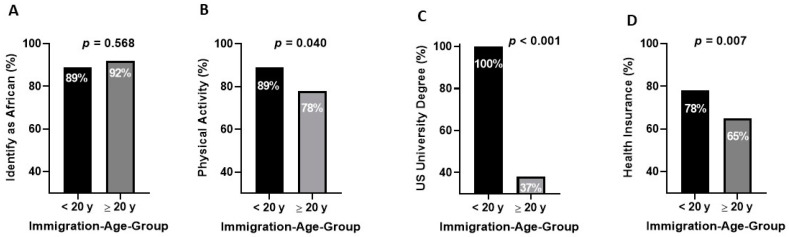
Comparison by Immigration-Age-Group of Key Cultural and Socioeconomic Factors. Black columns represent the Immigration-Age < 20 y group. Grey columns represent the Immigration-Age ≥ 20 y group. (**A**): Self Identify as African; (**B**): Physical Activity; (**C**): Degree from a university in the United States; (**D**): Health Insurance.

For the overall cohort, the rate at which participants self-identified as African was very high and did not differ by immigration age group (89% vs. 92%, *p* = 0.568) (Figure 3A).

When the overall cohort Immigration-Age < 20 y group was subdivided into two groups: <10 years and 10 to 19 years, the rate of African self-identification was lower in the <10 years group than the 10 to 19 years group (78% vs. 95%, *p* = 0.035) (Table 2). However, the majority of both groups self-identified as African.

### 3.4. Marital Status

Among those who were married, the rate of marriage to an African-born spouse was lower in the Immigration-Age < 20 y group than the Immigration-Age ≥ 20 y group (56% vs. 80%, *p* = 0.026) (Table 1). However, more than 50% of married African immigrants in both groups had African-born spouses. 

### 3.5. Physical Activity, Smoking and Alcohol Intake

The Immigration-Age < 20 y group had a higher physical activity rate than the Immigration-Age ≥ 20 y group (89% vs. 78%, *p* = 0.040) (Figure 3B).

Smoking and alcohol intake did not differ by immigration age group (Table 1).

### 3.6. Socioeconomic Status

Socioeconomic status as determined by income and college graduation rate were similar in the Immigration-Age < 20 y and Immigration-Age ≥ 20 y groups. Yet, one hundred percent of the Immigration-Age < 20 y group with college degrees graduated from an American University, compared to only 37% for the Immigration-Age ≥ 20 y (*p* < 0.001) (Figure 3C). The majority of both groups had health insurance, but health insurance coverage was more common in the Immigration-Age < 20 y group than the Immigration-Age ≥ 20 y group (78% vs. 65%, *p* = 0.007) (Figure 3D).

When the Immigration-Age < 20 y group was divided into two groups (i.e., Immigration-Age < 10 y and Immigration-Age-10-to-19-y), there was no difference between groups in income, college graduation rate, or health insurance (all *p* > 0.7) (Table 2).

### 3.7. The Combined Effect of Demographic, Socioeconomic, and Behavioral Factors on the CVD-Risk

Multiple regressions were performed with CVD-Risk as the dependent variable and immigration age, duration of US residence, education, health insurance, income, and alcohol intake as independent variables. As smoking is a component of the CVD-Risk, it was not included in the regression models. As physical activity data were only available in 56% of participants, it was not included.

For the Immigration-Age < 20 y group, adjusted R^2^ was 31%. Only two variables significantly contributed to the overall model, age at immigration, and US residence for ≥20 years (both *p* < 0.001) (Table 3).

**Figure 4:**
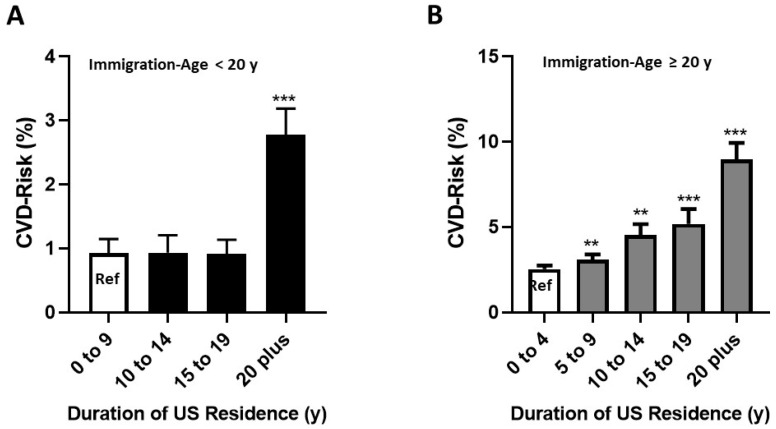
CVD-Risk by Duration of Residence in the United States for each Immigration-Age-Group. For both figures the first column is the reference group. ** *p* < 0.01, *** *p* < 0.001. (**A**): Immigration-Age < 20 y, (**B**): Immigration-Age ≥ 20 y.

The duration of United States residence intervals of 10 to 14 y and 15 to 19 y did not contribute to the CVD-Risk (Figure 4A).

For the Immigration-Age ≥ 20 y group, adjusted R^2^ was 41% (Table 4). The significant variables with positive β-coefficients were age at immigration, all 5 y increments of duration of residence in the United States (Figure 4B), and alcohol intake (all *p* < 0.001). The one significant variable with negative β-coefficient was income (*p* = 0.005).

## 4. Discussion

To our knowledge, this is the first investigation to compare the cardiometabolic health status of African immigrants who came to the United States before age 20 years versus after age 20 years. We found that independent of francophone status or African region of origin (Supplement Tables S1–S4), African immigrants who had arrived in the United States before the age of 20 had similar or better cardiometabolic health than immigrants who arrived in the United States at age 20 or older (Table 1). Based on the metabolic, behavioral, and socioeconomic data collected, the cardiometabolic health of African immigrants who came to the United States before age 20 appears to be positively influenced by maintenance of African identity and the actualization of the socioeconomic advantages that come with immigration before adulthood. Furthermore, we sub-divided the Immigration-Age < 20 y group into two smaller age groups: before age nine and between age 10 and 19 years (Table 2). Our findings of a high degree of cultural identification, as demonstrated by self-identifying as African, and optimization of opportunity applied to both groups.

Evidence for cultural retention in the Immigration-Age < 20 y group are provided by the observations that the majority of African immigrants who came to the United States before the age of 20 y identified as African (Figure 3A), married African-born spouses, and did not acquire adverse health behaviors, such as smoking or drinking, at rates higher than immigrants who came to the United States as adults. 

Home culture retention and family support in immigrant and indigenous communities are closely entwined [24]. Evidence linking cultural continuity in childhood to lower levels of stress, resilient behavior, and less cardiometabolic disease in adulthood was powerfully presented in a study by Currie et al. of indigenous adults living in Western Canada [24]. The value of family support on mitigating adverse cardiometabolic health was also evident in our earlier investigations in which we reported that immigrants who came to the United States for family reunification had lower allostatic loads than immigrants who were refugees or seeking asylum [3].

Furthermore, African immigrants who arrived as children may acquire behaviors in America that lead to improved health. African immigrants who arrived in America < 20 years of age were more physically active in adulthood than immigrants who came as adults (Figure 3B). In men, this difference in physical activity level had a positive and potentially important physiological manifestation. HDL levels are known to increase with exercise and decrease CVD risk [25]. Even after adjusting for age, HDL and the associated apolipoprotein (apoA1) were higher in the immigrants who came before the age of 20 y. Interestingly, Evenson et al. reported that Latina immigrants who arrived in the United States before the age of 25 engaged in more physical activity in adulthood than Latina immigrants who arrived when they were older than 25 years [26].

Evidence for realizing opportunity in America by the Immigration-age < 20 y group is demonstrated by the fact that 100% of the individuals who were college graduates in this group had obtained degrees from American universities compared to only 37% of college graduates in the Immigration-Age ≥ 20 y group. Compared to degrees obtained in Africa, Asia, or Europe, a degree from an American university may increase the opportunity for employment in the United States, which is commensurate with educational attainment. In addition, the benefit of health insurance is often linked to type of employment. Consistent with the concept that an American education and type of employment provides better health coverage, we found that immigrants who arrived before the age of 20 y had health insurance at a higher rate than immigrants who arrived in the United States after age 20 (Figure 3D).

### 4.1. CVD-Risk

The multiple regression analyses revealed that the factors that contributed to an elevated CVD-Risk in the Immigration-Age < 20 y group were age of immigration and more than 20 years residence in the United States (Table 3). For the Immigration-Age ≥ 20 y group, the factors contributing to increased CVD-Risk were age of immigration, income, alcohol intake, and more than five years residence in the United States.

#### 4.1.1. Education and Income

CVD-Risk was not influenced by education and income in the Immigration-Age < 20 y group. It is likely that neither a college education nor a higher income lowered CVD-Risk in the Immigration-Age < 20 y group for the same reasons they do not contribute to lowering allostatic load in African-Americans. Please note that comparing the Immigration-Age < 20 y group to African-Americans is fundamentally different than comparing the two immigration-age groups. After analyzing of National Health and Nutrition Examination Survey (NHANES) Data IV, Geronimus et al. reported that increased income in whites but not African-Americans led to a decrease in allostatic load [27]. Geronimus et al. attributed the inability of higher income to lower allostatic load score in African-Americans to the physiological consequence of coping with racism in America, a phenomenon the authors referred to as weathering [27]. In short, African immigrants who come to the United States as children will have life-time exposure to the same pressures and prejudices that African-Americans face.

In contrast, higher income in the Immigration-age ≥ 20 y group was associated with a lower CVD-Risk. We speculate that the difference in the relationship between income and CVD-Risk Score in the two immigration age groups is related to remittances. For decades, the sending of money by immigrants to relatives in Africa has been considered a familial obligation of such magnitude that it represents a major form of international trade. According to the World Bank, in 2018, Nigerians received 24.2 billion dollars from overseas Nigerians, and this represented 6.1% of Nigeria’s gross domestic product (GDP) [28]. In the same time frame, Ghanaian immigrants sent 3.8 billion dollars to relatives in Ghana, and this represented 7.3% of Ghana’s GDP [28]. The economic challenge of sending money to relatives in Africa is a major source of stress for African immigrants [29]. It is conceivable that a higher income may decrease the physiological stress induced by this financial obligation and decrease the risk of heart disease [30]. We did not collect data on remittance patterns, but we suspect that sending remittances to Africa is more common in the Immigration-Age ≥ 20 y group than the Immigration-Age < 20 y group.

#### 4.1.2. Alcohol Intake

Alcohol intake promoted CVD-risk in the Immigration-Age ≥ 20 y group, but not the Immigration-Age < 20 y group. It is possible that the Immigration-Age ≥ 20 y group consumes more alcohol per week than the Immigration-Age < 20 y group. Our question about alcohol consumption inquires only whether a person has one or more drinks per week. Information on the upper limit is not requested. Our finding that greater alcohol consumption is associated with increased CVD in the immigration-Age ≥ 20 group is consistent with the concept that immigration as an adult is stressful [31,32]. Stress promotes both cardiovascular disease and alcohol intake with the latter prompting the former [33]. Overall, we believe that members of the Immigration-Age ≥ 20 y group immigrate more frequently in isolation and may have less social support than immigrants who arrive as children with their families.

#### 4.1.3. Duration of Residence

Duration of stay in the United States was longer for the Immigration-Age < 20 y group than the Immigration-Age ≥ 20 y group (Figure 2), but the effect of duration of residence in the United States on the CVD-Risk was markedly different. For the Immigration-Age < 20 y group, there was no effect of duration of residence on CVD-Risk until the stay in the United States was ≥20 years (Figure 4A, Table 3). In a prospective analyses relating diabetes in adulthood to age of immigration in the foreign-born adults enrolled in the National Health Interview Survey Oza-Frank et al. also reported that for childhood immigrants the first two decades of life in the United States did not contribute to diabetes risk in adulthood [34].

In short, the first two decades of life in the United States do not appear to adversely affect the rate of heart disease in African immigrants who came to the United States as children.

However, the effect of duration of United States residence on CVD-Risk is different for adult immigrants. For the Immigration-Age ≥ 20 y group, every 5 y increment (5 to 9 y, 10 to 14 y, 15 to 19 y and 20 y plus) was associated with a highly significant increase in CVD-Risk. Again, Oza-Frank et al. had a similar finding for diabetes risk in foreign-born adults who came to the United States after age 18 [34].

Based on the findings of Oza-Frank et al. with diabetes and ours with CVD-Risk, we suggest that when duration of stay is related to immigrant health, age of immigration should be considered as a dichotomous variable (i.e., before or after age 20 y) [34]. If this is not done or not possible because of a lack of data on immigration age, conclusions about the effect of the first 20 years of residence in the United States on the risk for either CVD or diabetes will be confounded.

### 4.2. Diverse Immigrant Populations

Among the foreign-born populations in the United States, there is great diversity in ethnic identity, genetic background, reason for immigration, and the sociocultural, economic, and political realities of daily life. For many reasons, including differences in body mass index across population groups, our conclusions about the effect of age of immigration on the cardiovascular health of African immigrants may not be applicable to other immigrant groups of African descent such as Blacks from the Caribbean, United Kingdom, France, or Canada [35,36]. In addition, as our findings about African immigrant health are fundamentally different from reports in Hispanic and Asian immigrants [5,6,8,11,12,13], it is essential to not generalize about cardiometabolic health across immigrant populations.

### 4.3. Strengths and Limitations

The limitations of this study include the cross-sectional design, the lack of dietary data, the size of the cohort, and the use of a convenience sample. Nonetheless, there are characteristics of the cohort that give us confidence in both the size of the sample and its representative nature. Consistent with known immigration patterns, the majority of the participants were from non-francophone countries in West Africa [1]. Washington, DC, is a top settlement area for African immigrants in the United States [1]. In addition, our recruitment area included two of the five counties in the United States with the highest concentration of African-born Blacks, specifically Montgomery County, Maryland, and Prince George’s County, Maryland [1]. Furthermore, and as would be expected in a representative sample, sickle cell trait and hemoglobin C were more common in West and Central Africans than East Africans [37,38]. The gender distribution of the cohort also followed expected patterns. The majority of immigrants who come from Africa to the United States as adults were male [1]. However, in the Immigrant-Age < 20 y group, 53% were male, which is consistent with the anticipated male–female ratio when families immigrate. In addition, the prevalence of diabetes in our cohort was 7%, which is similar to the 9% prevalence of diabetes reported in African immigrants in Canada [39]. Equivalent data are not available in the United States.

However, about behaviors that increase cardiovascular risk, specifically smoking, we documented that only 5% of African immigrants smoked cigarettes, but we did not quantify the amount smoked. In addition, while we had educational data on all participants, we only collected information on location of degree granting institution in the most recently enrolled 74 participants.

## 5. Conclusions

African immigrants who came to the United States before the age of 20 years have, in adulthood, similar or even better cardiometabolic health than immigrants who arrived at 20 years or older. The likely reasons for why African immigrants who come to the United States age < 20 y maintain their cardiometabolic health into adulthood are a strong sense of family and cultural identity; the failure to acquire adverse health behaviors; the adoption of positive behaviors, such as exercise; and the ability to optimize opportunities in America such as achieving an American college education and an occupation that provides health insurance. The health of immigrants is most often analyzed according to duration of residence in the United States. Yet our data suggests that the first 10 to 20 years of residence may have adverse health effects only on the African immigrants who came to the United States as adults. Going forward, we recommend analyzing the health of African immigrants from the perspective of age of immigration.

## Figures and Tables

**Table 1:** Characteristics by Immigration Age Group.

Parameters ^1^	Total *n* = 482 100%	Immigration Age Less than 20 y *n* = 111 23%	Immigration Age 20 to 65 y *n* = 371 77%	*p*-Value ^2^
Current Age (y) ^3^	38 ± 1	31 ± 1	40 ± 1	<0.001
Age at immigration (y)	26 ± 1	12 ± 1	31 ± 1	<0.001
Duration of Residence in US (y)	12 ± 1	24 ± 1	8 ± 1	<0.001
Male (%)	65%	53%	69%	0.003
Obesity (%)	27%	23%	29%	0.209
Diabetes (%) ^3^	7%	4%	8%	0.123
CVD-Risk (%)	3.69 ± 0.21	3.94 ± 0.35	3.62 ± 0.18	0.413
BMI (kg/m^2^)	27.6 ± 0.2	27.6 ± 0.4	27.6 ± 0.2	0.962
WC (cm)	90.7 ± 0.5	90.5 ± 1.1	90.8 ± 0.6	0.818
Systolic BP (mmHg) ^3^	120 ± 1	119 ± 1	120 ± 1	0.410
Diastolic BP (mmHg)	72 ± 1	72 ± 1	72 ± 1	0.664
Pulse (beat/min)	67 ± 1	68 ± 1	67 ± 1	0.910
Cholesterol (mg/dL) ^3^	164 ± 2	164 ± 3	164 ± 2	0.778
HDL (mg/dL) (male) ^3^	50 ± 1	52 ± 2	47 ± 1	0.014
HDL (mg/dL) (female) ^3^	57 ± 1	59 ± 2	57 ± 1	0.382
TG (mg/dL)	73 ± 2	67 ± 4	75 ± 2	0.062
LDL (mg/dL)	98 ± 1	96 ± 3	99 ± 2	0.486
apoA1 (mg/dL) (male)	129 ± 1	135 ± 3	128 ± 1	0.015
apoA1 (mg/dL) (female)	141 ± 2	144 ± 3	140 ± 2	0.260
apoB (mg/dL)	82 ± 1	81 ± 2	83 ± 1	0.589
Identify as African (*n* = 255)	91%	89%	92%	0.568
Partnered (%) ^4^	48%	29%	54%	<0.001
African-born Partner (%) (*n* = 113)	76%	56%	80%	0.026
Physical Activity (*n* = 270) ^5^	81%	89%	78%	0.040
Smoker ^3^	5%	5%	5%	0.814
Alcohol ≥ 1 drink/week	26%	32%	24%	0.111
Income ≥ 40 k	48%	53%	47%	0.228
College Graduate (%)	72%	67%	74%	0.154
American University Degree (*n* = 74)	61%	100%	37%	<0.001
Health Insurance (%)	68%	78%	65%	0.007

^1^ Data presented as mean ± SE or percentages. ^2^ For continuous variables, multiple regression were done to adjust for age, for categorical variables chi-square analyses were performed. ^3^ Variables used to calculate CVD-Risk. ^4^ Married or living with partner (alternative category: never married, separated, divorced, widowed). ^5^ IPAQ Category (Moderate or High) vs. (Low).

**Table 2:** Characteristics of the Immigration-Age < 20 y Group.

Parameters ^1^	Total Less than 20 y *n* = 111 100%	Immigration Age Less than 10 y *n* = 44 40%	Immigration Age 10 to 19 y *n* = 67 60%	*p*-Value ^2^
Current Age (y) ^3^	31 ± 1	30 ± 1	31 ± 1	0.576
Age at Immigration (y)	12 ± 1	5 ± 1	16 ± 1	<0.001
Duration of Residence in US (y)	19 ± 1	25 ± 1	16 ± 1	<0.001
Male (%)	53%	50%	55%	0.590
Obesity (%)	23%	25%	21%	0.613
Diabetes (%) ^3^	5%	5%	5%	0.987
CVD-Risk (%)	1.69 ± 0.21	1.51 ± 0.22	1.82 ± 0.31	0.463
BMI (kg/m^2^)	26.8 ± 0.4	27.0 ± 0.7	26.7 ± 0.20.5	0.742
WC (cm)	87.2 ± 1.3	87.5 ± 2.1	87.0 ± 1.6	0.865
Systolic BP (mmHg) ^3^	117 ± 1	116 ± 2	117 ± 1	0.718
Diastolic BP (mmHg)	70 ± 1	70 ± 1	70 ± 1	0.917
Pulse (beat/min)	67 ± 1	66 ± 1	68 ± 1	0.287
Cholesterol (mg/dL) ^3^	157 ± 3	162 ± 5	153 ± 4	0.178
HDL (mg/dL) ^3^ male	51 ± 2	49 ± 3	52 ± 2	0.413
HDL (mg/dL) ^3^ female	57 ± 2	57 ± 2	57 ± 2	0.856
TG (mg/dL)	62 ± 3	67 ± 5	58 ± 3	0.122
LDL (mg/dL)	91 ± 3	96 ± 5	87 ± 4	0.166
apoA1 (md/dL) male	132 ± 2	130 ± 4	134 ± 3	0.413
apoA1 (mg/dL) female	141 ± 3	141 ± 5	140 ± 4	0.835
apoB (mg/dL)	76 ± 2	81 ± 4	73 ± 3	0.090
Identify as African (*n* = 65)	89%	78%	95%	0.035
Partnered (%) ^4^	29%	16%	37%	0.015
African spouse (%) (*n* = 18)	56%	50%	57%	0.800
Smoker ^3^	5%	7%	5%	0.599
Alcohol ≥ 1 drink/week	32%	34%	30%	0.403
Physical Activity (*n* = 66) ^5^	89%	100%	84%	0.041
Income ≥ 40 k	53%	52%	54%	0.809
College Graduate (%)	67%	68%	66%	0.784
American Univ Degree (*n* = 27)	100%	100%	100%	0.999
Health Insurance (%)	78%	80%	78%	0.809

^1^ Data presented as mean ± SE or percentages. ^2^ Chi-square or unpaired *t*-test. ^3^ Variables used to calculate CVD-Risk. ^4^ Married or living with partner (category includes: never married, separated, divorced, widowed). ^5^ IPAQ Category (Moderate or High) vs. (Low).

**Table 3:** Multiple Regression with CVD-Risk as the Dependent Variable in the Immigration-Age < 20 y Group. Adjusted R^2^: 31% (*n* = 110) ^1^.

Independent Variable	β-Coefficient	SE	*p*-Value
Immigration Age	0.200	0.039	<0.001
US residence 10 to 14 y ^2^	0.581	0.657	0.379
US residence 15 to 19 y ^2^	0.880	0.565	0.123
US residence 20 y plus ^2^	3.620	0.624	<0.001
Education	−0.145	0.159	0.366
Health Insurance	−0.158	0.438	0.719
Income	−0.044	0.110	0.687
Alcohol Intake	−0.147	0.387	0.706

^1^ One participant had missing data on duration of US residence. ^2^ Reference group is US residence 0 to 9 y.

**Table 4:** Multiple Regression with CVD-Risk as the Dependent Variable in the Immigration-Age ≥ 20 y Group. Adjusted R^2^: 41% (*n* = 368) ^1^.

Independent Variable	β-Coefficient	SE	*p*-Value
Immigration Age	0.271	0.027	<0.001
US residence 5 to 9 y ^2^	1.874	0.570	0.001
US residence 10 to 14 y ^2^	2.548	0.643	<0.001
US residence 15 to 19 y ^2^	5.275	0.752	<0.001
US residence 20 y plus ^2^	8.609	0.652	<0.001
Education	0.180	0.161	0.262
Health Insurance	0.238	0.460	0.606
Income	−0.323	0.115	0.005
Alcohol Intake	2.237	0.457	<0.001

^1^ Three participants had missing data on duration of US residence. ^2^ Reference group is US residence 0 to 4 y.

## Supplementary Materials

The following are available online at https://www.mdpi.com/1660-4601/17/24/9405/s1, Table S1: Characteristics by African Region of Origin, Table S2: Characteristics According to Francophone Status of Country of Origin, Table S3: Characteristics by Immigration-Age-Group of Participants from Francophone Countries, Table S4: Characteristics by Immigration-Age-Group of Participants from Non-Francophone Countries.
