# Supplementary material for: Africans Who Arrive in the United States before 20 Years of Age Maintain Both Cardiometabolic Health and Cultural Identity: Insight from the Africans in America Study
Source: Int J Environ Res Public Health. 2020 Dec 15;17(24):9405. doi: 10.3390/ijerph17249405 (PMC7765413; doi:10.3390/ijerph17249405)
Supplement: Supplementary file 1 [file ijerph-17-09405-s001.pdf]

**Table S1: Characteristics by African Region of Origin**

| Parameters[1] | West 52% n=253 | Central[2] 20% n=96 | East 28% n=133 | *P*-value[3] |
|---|---|---|---|---|
| **Current Age (y)** | 39±1 | 37±1 | 38±1 | 0.430 |
| **Age at Immigration (y)** | 25±1 | 28±10 | 27±1 | 0.125 |
| **Male (%)** | 64% | 73% | 62% | 0.174 |
| **Identify as African (n=255)** | 90% | 87% | 94% | 0.377 |
| **Duration of Residence in US (y)** | 14±11 | 10±1 | 11±1 | 0.002, a**, b* |
| **Partnered (%)[4]** | 45% | 48% | 53% | 0.298 |
| **African-born partner (%) (n=113)** | 67% | 77% | 87% | 0.072 |
| **Smoker (%)** | 5% | 4% | 5% | 0.918 |
| **Alcohol ≥ 1drink/week** | 24% | 25% | 29% | 0.529 |
| **Physical Activity (n=270)[5]** | 80% | 82% | 81% | 0.950 |
| **Health Insurance (%)** | 68% | 66% | 69% | 0.849 |
| **College Graduate (%)** | 75% | 66% | 71% | 0.196 |
| **Income ≥40k** | 51% | 37% | 50% | 0.037 |
| **Hemoglobin (g/dL)** | 13.9±0.1 | 14.0±0.1 | 14.2±0.1 | 0.228 |
| **Hematocrit (%)** | 41.7±0.3 | 41.6±0.4 | 42.2±0.3 | 0.338 |
| **Sickle Cell Trait** | 16% | 19% | 8% | 0.013 |
| **Hemoglobin C Trait** | 6% | 0% | 0% | 0.013 |
| **Obesity (%)** | 30% | 26% | 23% | 0.280 |
| **Diabetes (%)** | 7% | 9% | 8% | 0.768 |
| **CVD-Risk (%)** | 4.00±0.31 | 4.08±0.54 | 2.96±0.27 | 0.075 |

[1] Data presented as mean±SE or percentages
[2] The four individuals from southern African countries were analyzed with the Central Africa group
[3] Comparisons were by one-way ANOVA with Bonferroni corrections (a: West vs. Central, b: West vs East, c: Central vs. East, *$P$≤0.05, **$P$≤0.01, ***$P$≤0.001) or chi-square as appropriate.
[4] Married or living with partner (alternative: never married, separated, divorced, widowed)
[5] IPAQ Category (Moderate or High) vs (Low)

**Table S2: Characteristics According to Francophone Status of Country of Origin**

| Parameters[1] | Total Cohort 100% n=482 | Francophone 34% n=162 | Non-Francophone 66% n=320 | *P*-value[2] |
|---|---|---|---|---|
| **Current Age (y)[3]** | 38±1 | 38±1 | 38±1 | 0.598 |
| **Age at Immigration (y)** | 26±1 | 29±1 | 25±1 | 0.002 |
| **Duration of Residence in US (y)** | 12±1 | 10±1 | 13±1 | <0.001 |
| **Male (%)** | 65% | 69% | 63% | 0.241 |
| **Obesity (%)** | 27% | 27% | 28% | 0.824 |
| **Diabetes (%)[3]** | 8% | 7% | 8% | 0.526 |
| **CVD-Risk (%)** | 3.73±0.21 | 3.58±0.33 | 3.81±0.27 | 0.604 |
| **BMI (kg/m$^2$)** | 27.6±0.20 | 27.4±0.36 | 27.8±0.25 | 0.436 |
| **WC (cm)** | 91±1 | 90±1 | 91±1 | 0.285 |
| **Systolic BP (mmHg)[3]** | 120±1 | 121±1 | 119±1 | 0.248 |
| **Diastolic BP (mmHg)** | 72±0.4 | 73±0.8 | 72±0.5 | 0.253 |
| **Pulse (beats/min)** | 67±0.4 | 67±0.8 | 68±0.5 | 0.293 |
| **Cholesterol (mg/dL)[3]** | 164±1.6 | 160±2.8 | 166±1.9 | 0.091 |
| **TG (mg/dL)** | 73±2 | 70±3 | 75±2 | 0.152 |
| **HDL (mg/dL)[3]** | 51±1 | 51±1 | 51±1 | 0.955 |
| **LDL (mg/dL)** | 98±1 | 95±3 | 100±2 | 0.151 |
| **apoA1 (mg/dL)** | 133±1 | 133±1 | 134±2 | 0.624 |
| **apoB (mg/dL)** | 82±1 | 79±2 | 84±1 | 0.041 |
| **Identify as African (n=255)** | 91% | 91% | 92% | 0.683 |
| **Partnered (%) [4]** | 48% | 52% | 46% | 0.220 |
| **African-born Partner (%) (n=113)** | 76% | 86% | 70% | 0.052 |
| **Physical Activity (n=270)[5]** | 81% | 82% | 80% | 0.728 |
| **Smoker (%)[3]** | 5% | 5% | 5% | 0.977 |
| **Alcohol ≥ 1drink/week** | 26% | 23% | 27% | 0.302 |
| **Income ≥40k** | 48% | 44% | 50% | 0.249 |
| **College Graduate (%)** | 72% | 70% | 73% | 0.426 |
| **American University Degree (n=74)** | 61% | 50% | 64% | 0.280 |
| **Health Insurance (%)** | 68% | 66% | 69% | 0.549 |

[1] Data presented as mean±SE or percentages 2 2
[2] Comparisons were unpaired t-tests or chi-square as appropriate.
[3] Variables used to calculate CVD-Risk
[4] Married or living with partner (alternative: never married, separated, divorced, widowed)
[5] IPAQ Category (Moderate or High) vs (Low)

**Table S3: Characteristics by Immigration-Age-Group of Participants from Francophone Countries**

| Parameters[1] | Total 100% n=162 | Immigration Age Less than 20y n=19 | Immigration Age 20 to 65y n=143 | *P*-value[2] |
|---|---|---|---|---|
| **Current Age (y)[3]** | 38±1 | 27±1 | 39±1 | <0.001 |
| **Age at Immigration** | 29±1 | 14±1 | 31±1 | <0.001 |
| **Duration of Residence in US (y)** | 10±1 | 14±2 | 9±1 | <0.001 |
| **Male (%)** | 69% | 53% | 71% | 0.113 |
| **Obesity (%)** | 27% | 26% | 27% | 0.981 |
| **Diabetes (%)[3]** | 7% | 5% | 7% | 0.778 |
| **CVD-Risk (%)** | 3.58±0.33 | 4.20±0.78 | 3.49±0.27 | 0.403 |
| **BMI (kg/m$^2$)** | 27.4±0.4 | 27.5±1.1 | 27.4±0.4 | 0.957 |
| **WC (cm)** | 89.9±0.9 | 90.4±2.7 | 89.8±0.9 | 0.829 |
| **Systolic BP (mmHg)[3]** | 121±1 | 122±3 | 120±1 | 0.599 |
| **Diastolic BP (mmHg)** | 73±1 | 75±2 | 73±1 | 0.258 |
| **Pulse (beat/min)** | 67±1 | 71±3 | 66±1 | 0.077 |
| **Cholesterol (mg/dL) [3]** | 160±3 | 153±8 | 162±3 | 0.287 |
| **HDL (mg/dL) (male)[3]** | 49±1 | 52±4 | 49±1 | 0.503 |
| **HDL (mg/dL) (female)[3]** | 55±2 | 61±5 | 54±2 | 0.214 |
| **TG (mg/dL)** | 70±3 | 61±8 | 71±3 | 0.252 |
| **LDL (mg/dL)** | 95±3 | 85±7 | 97±2 | 0.129 |
| **apoA1 (mg/dL) (male)** | 130±2 | 132±7 | 130±1 | 0.728 |
| **apoA1 (mg/dL) (female)** | 143±4 | 148±10 | 142±4 | 0.589 |
| **apoB (mg/dL)** | 82±1 | 81±2 | 83±1 | 0.589 |
| **Identify as African (n=76)** | 92% | 100% | 91% | 0.416 |
| **Partnered (%)[4]** | 48% | 21% | 56% | 0.004 |
| **African-born partner (%) (n=43)** | 86% | 67% | 88% | 0.315 |
| **Physical Activity (n=78)[5]** | 80% | 100% | 80% | 0.195 |
| **Smoker[3]** | 5% | 0% | 6% | 0.290 |
| **Alcohol ≥ 1drink/week** | 23% | 32% | 22% | 0.334 |
| **Income ≥40k** | 44% | 37% | 46% | 0.478 |
| **College Graduate (%)** | 70% | 63% | 71% | 0.505 |
| **American University Degree (n=18)** | 50% | 100% | 40% | 0.058 |
| **Health Insurance (%)** | 66% | 63% | 66% | 0.777 |

[1] Data presented as mean±SE or percentages
[2] Chi-square or multiple regression adjusting for age
[3] Variables used to calculate CVD-Risk
[4] Married or living with partner (alternative: never married, separated, divorced, widowed)
[5] IPAQ Category (Moderate or High) vs (Low)

**Table S4: Characteristics by Immigration-Age-Group of Participants from Non-Francophone Countries**

| Parameters[1] | Total 100% n=320 | Immigration Age Less than 20y n=92 | Immigration Age 20 to 65y n=228 | *P*-value[2] |
|---|---|---|---|---|
| **Current Age (y)[3]** | 38±1 | 32±1 | 41±1 | <0.001 |
| **Age at Immigration** | 25±1 | 11±1 | 31±1 | <0.001 |
| **Duration of Residence in US (y)** | 13±1 | 20±1 | 10±1 | <0.001 |
| **Male (%)** | 63% | 53% | 67% | 0.020 |
| **Obesity (%)** | 28% | 22% | 30% | 0.143 |
| **Diabetes (%)[3]** | 8% | 4% | 10% | 0.095 |
| **CVD-Risk (%)** | 3.81±0.35 | 3.99±0.41 | 3.73±0.25 | 0.607 |
| **BMI (kg/m$^2$)** | 27.8±0.2 | 27.7±0.5 | 27.8±0.3 | 0.858 |
| **WC (cm)** | 91.1±0.7 | 90.5±1.2 | 91.3±0.7 | 0.599 |
| **Systolic BP (mmHg)[3]** | 119±1 | 118±2 | 120±1 | 0.361 |
| **Diastolic BP (mmHg)** | 72±1 | 71±1 | 72±1 | 0.372 |
| **Pulse (beat/min)** | 68±1 | 67±1 | 68±1 | 0.306 |
| **Cholesterol (mg/dL)[3]** | 166±2 | 167±4 | 166±2 | 0.857 |
| **HDL (mg/dL) (male)[3]** | 47±1 | 52±2 | 46±1 | 0.005 |
| **HDL (mg/dL) (female)[3]** | 58±1 | 58±2 | 58±1 | 0.943 |
| **TG (mg/dL)** | 75±2 | 68±4 | 78±3 | 0.054 |
| **LDL (mg/dL)** | 100±2 | 98±3 | 101±2 | 0.532 |
| **apoA1 (mg/dL) (male)** | 129±1 | 136±3 | 126±1 | 0.004 |
| **apoA1 (mg/dL) (female)** | 140±2 | 144±4 | 138±2 | 0.226 |
| **apoB (mg/dL)** | 84±1 | 83±2 | 84±1 | 0.643 |
| **Identify as African (n=179)** | 91% | 88% | 92% | 0.416 |
| **Partnered (%)[4]** | 46% | 30% | 52% | <0.001 |
| **African-born Partner (%) (n=70)** | 70% | 53% | 75% | 0.112 |
| **Exercise (n=192)[5]** | 80% | 88% | 77% | 0.066 |
| **Smoker[3]** | 5% | 7% | 4% | 0.428 |
| **Alcohol ≥ 1drink/week** | 27% | 32% | 25% | 0.268 |
| **Income ≥40k** | 50% | 57% | 47% | 0.138 |
| **College Graduate (%)** | 73% | 67% | 75% | 0.142 |
| **American Univ Degree (n=56)** | 61% | 100% | 38% | <0.001 |
| **Health Insurance (%)** | 69% | 82% | 64% | 0.002 |

[1] Data presented as mean±SE or percentages
[2] Chi-square or multiple regression adjusting for age
[3] Variables used to calculate CVD-Risk
[4] Married or living with partner (alternative: never married, separated, divorced, widowed)
[5] IPAQ Category (Moderate or High) vs (Low)
